# Better together: the transcription factor MUTE aggregates into nuclear condensates to regulate stomatal development in maize

**DOI:** 10.1093/plcell/koag050

**Published:** 2026-02-28

**Authors:** Pablo González-Suárez

**Affiliations:** Assistant Features Editor, the Plant Cell, American Society of Plant Biologists; Department of Developmental Genetics, Centre for Plant Molecular Biology (ZMBP), Eberhard Karls University, Tuebingen D-72076, Germany

Moving from water to land was no easy job for plants. Over millions of years, ancestors of modern land flora evolved critical adaptations to prevent desiccation and enable life on land. Among these traits are stomata, pores in the epidermis that mediate water and gas exchange with the atmosphere. The genetic regulation of stomatal development is remarkably conserved across the plant kingdom and relies primarily on basic helix-loop-helix (bHLH) transcription factors. Over time, however, these proteins have evolved and subfunctionalized to enable the diversity of stomatal forms observed across different species (Spiegelhalder and Raissig 2021) (Fig. 1).

**Figure 1:**
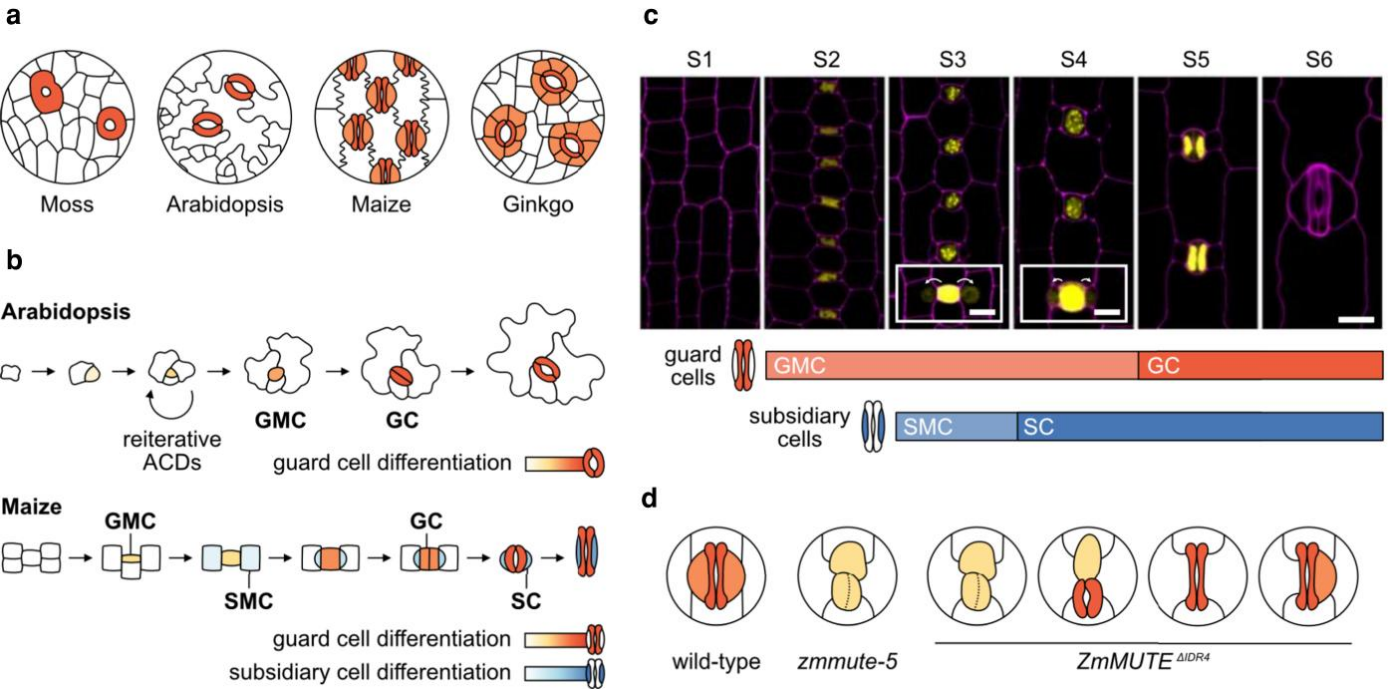
Nuclear condensates of ZmMUTE regulate guard cell and subsidiary cell development in maize. a) Diagrams illustrating morphological diversity of stomatal complexes across different species: moss (*Physcomitrium patens*), Arabidopsis (*Arabidopsis thaliana*), maize (*Zea mays*) and ginkgo (*Ginkgo biloba*). b) Comparative cartoon of stomatal development in Arabidopsis and maize. c) Timeline of ZmMUTE protein dynamics (*ZmMUTEp:Venus-3Myc-ZmMUTE*) from Qiao et al. (2026). For stages 3 and 4, the inserts include images with increased laser intensity highlighting ZmMUTE movement to SMCs and SCs. The diagrams below the confocal images indicate the approximate times at which GMCs, GCs, SMCs, and SCs are formed. d) Drawings illustrating main phenotypes of the strong *zmmute-5* loss-of-function allele and the *ZmMUTE* variant with a disrupted IDR4 (*ZmMUTE^ΔIDR4^*). ACD, asymmetric cell division. Figure credit: P. González-Suárez.

In the model dicot Arabidopsis, stomata form through a series of asymmetric divisions of a precursor cell, named meristemoid, which eventually acquires the identity of a guard mother cell (GMC). The GMC then divides symmetrically to produce a pair of kidney-shaped guard cells (GCs). The homeologs SPEECHLESS, MUTE, and FAMA orchestrate sequential steps in this developmental sequence by forming bHLH transcription factor heterodimers with their partners SCREAM and SCREAM2. In contrast, monocot stomata feature dumbbell-shaped GCs flanked by 2 subsidiary cells (SCs) (Fig. 1), an innovation that improves physiological performance (Raissig et al. 2017). In this system, 2 sets of asymmetric divisions are needed: the first initiates the GMC from a protodermal cell, while the second recruits the subsidiary mother cells (SMCs) from neighboring cells. Recruitment of lateral SCs to the stomatal complex is unique to grasses, and the grass paralog of MUTE is a key factor involved in this process.

In dicots, MUTE is cell autonomous and is expressed in GMCs to halt reiterative asymmetric divisions of stomatal precursors. In grasses, however, MUTE is mobile and relocates from the GMC to the neighboring cells to specify SMC identity and promote their asymmetric division into daughter SCs (Raissig et al. 2017; Wang et al. 2019). The biochemical properties underlying these functional differences in MUTE between species and how MUTE from grasses integrates spatio-temporal signals to regulate gene expression have remained open questions.

Focusing on the stomatal apparatus of maize (*Zea mays*), recent work by Qiao and colleagues (Qiao et al. 2026) sheds new light on these matters. Their newly characterized *zmmute-5* mutant confirms previous observations that strong loss-of-function alleles of *ZmMUTE* impair asymmetric divisions in both GMCs and SMCs (Wang et al. 2019). Weak *zmmute-6* alleles, however, can produce GCs but rarely initiate SMCs, suggesting a more prominent role of *ZmMUTE* in SC formation. In line with this, overexpressing *ZmMUTE* leads to excessive divisions of SMCs and clusters of SCs with no apparent effects on GC development.

Through a combination of single-particle tracking and variable-angle epifluorescence microscopy, the authors next made an intriguing observation that, rather than uniformly dispersing within the nucleus, ZmMUTE forms aggregates. In vitro protein work suggests that these structures are membraneless condensates formed through liquid-liquid phase separation (LLPS), a property not observed in Arabidopsis's MUTE (AtMUTE), which neither moves nor aggregates. Interestingly, immunofluorescence assays demonstrated that these condensates co-localize with RNA polymerase II, suggesting that they are associated with transcriptionally active regions.

But what molecular features enable ZmMUTE to form these condensates, and how important are they for protein mobility? A closer look at its protein sequence revealed that ZmMUTE contains 4 intrinsically disordered regions (IDRs). Specifically, the authors found that IDR4 is key for aggregation and LLPS of the protein. ZmMUTE mutant variants lacking IDR4 (ZmMUTE^ΔIDR4^) fail to form condensates or move non–cell-autonomously and are, as a result, insufficient for proper SC development. Conversely, attaching IDR4 to AtMUTE was sufficient to confer the resulting chimeric protein mobility in both maize and in Arabidopsis. Even in the absence of IDR4, ZmMUTE interacts with ZmSCREAM and ZmSCREAM2. However, when IDR4 was removed, the transcriptional activation of ZmMUTE's targets was compromised, suggesting that condensation is required for ZmMUTE's function.

Conserved developmental regulators have subspecialized in different species to give rise to the rich morphological diversity found in angiosperms. Stomatal bHLH transcription factors provide a fascinating case study. In grasses, MUTE has neofunctionalized to recruit SCs to the 4-celled stomatal complex, acquiring the ability to move out of GMCs. Qiao et al. (2026) have provided compelling evidence that aggregation into nuclear condensates is important for MUTE's function and mobility in grasses, where it modulates the development of distinct stomatal cell types. Furthermore, they have found that grass-specific IDR4 confers ZmMUTE the ability to form condensates and move, raising interesting questions about the widespread role of LLPS in stomatal development in different plant species.

## Recent related articles in *The Plant Cell*

- Nan et al. (2023) showed that the myosin XI protein OPAQUE1 mediates phragmoplast guidance during asymmetric cell division of stomatal precursors in maize.
- Su et al. (2025) demonstrated that an intrinsically disordered region underpins the aggregation of SUPPRESSOR OF rps4-RLD1 during root development in Arabidopsis.
- Sun et al. (2022) used the maize single-nucleus transcriptome to investigate signaling networks underlying the movement and development of grass stomata.
- van Weringh et al. (2025) profiled guard cell–specific transcriptomes in Arabidopsis during drought to identify stress-responsive genes in the stomatal lineage.
- Wu et al. (2024) found that the MPK3/MPK6 mitogen-activated protein kinase cascade coordinates mesophyll and stomatal development in Arabidopsis.

## Data Availability

No new data were generated or analysed in support of this research.

## Dive Curated Terms

The following phenotypic, genotypic, and functional terms are of significance to the work described in this paper:

ZmMUTE Gramene: Zm00001d009778
ZmMUTE Araport: Zm00001d009778
